# Withdrawal of unnecessary antidepressant medication: a randomised controlled trial in primary care

**DOI:** 10.3399/bjgpopen17X101265

**Published:** 2017-11-15

**Authors:** Rhona Eveleigh, Esther Muskens, Peter Lucassen, Peter Verhaak, Jan Spijker, Chris van Weel, Richard Oude Voshaar, Anne Speckens

**Affiliations:** 1 Nursing Home Physician, Department of Primary and Community Care, Radboud University Medical Center, Nijmegen, The Netherlands; 2 Psychologist, Department of Primary and Community Care, Radboud University Medical Center, Nijmegen, The Netherlands; 3 GP, Senior Researcher, Department of Primary and Community Care, Radboud University Medical Center, Nijmegen, The Netherlands; 4 Professor of Primary Care, Department of Primary Care, University Groningen, University Medical Center Groningen, Nijmegen, The Netherlands; 5 Professor of Primary Care, Department of Mental Health, Netherlands Institute for Health Service Research (NIVEL), Utrecht, The Netherlands; 6 Professor of Psychiatry, Center of Depression Expertise, Pro Persona, Nijmegen, The Netherlands; 7 Professor of Psychiatry, Behavioural Science Institute, Radboud University Nijmegen, Nijmegen, The Netherlands; 8 Emeritus Professor of Primary Care, Department Health Services Research and Policy, Australian National University, Canberra, Australia; 9 Emeritus Professor of Primary Care, Department of Primary and Community Care, Radboud University Medical Center, Nijmegen, The Netherlands; 10 Professor of Psychiatry, Department of Psychiatry, Radboud University Nijmegen Medical Center, Nijmegen, The Netherlands; 11 Professor of Psychiatry, University Medical Center Groningen, University Center for Psychiatry & Interdisciplinary Center for Psychopathology of Emotion Regulation (ICPE), University of Groningen, Groningen, The Netherlands; 12 Professor of Psychiatry, Department of Psychiatry, Radboud University Nijmegen Medical Center, Nijmegen, The Netherlands

**Keywords:** antidepressant agents, primary care, depressive disorder, anxiety disorder, general practice, inappropriate prescribing

## Abstract

**Background:**

Antidepressant use has increased exponentially in recent decades, mostly due to long continuation.

**Aim:**

To assess the effectiveness of a tailored recommendation to withdraw antidepressant treatment.

**Design & setting:**

Randomised controlled trial in primary care (PANDA study) in the Netherlands.

**Method:**

Long-term antidepressant users (≥9 months) were selected from GPs prescription databases. Patients were diagnosed with the Composite International Diagnostic Interview (CIDI). Long-term users without indication for maintenance treatment (overtreatment) were selected. The intervention consisted of disclosure of the current psychiatric diagnosis combined with a tailored treatment recommendation. Patients were followed for 12 months.

**Results:**

The study included 146 participants from 45 family practices. Of the 70 patients in the intervention group, 34 (49%) did not comply with the advice to stop their antidepressant medication. Of the 36 (51%) patients who agreed to try, only 4 (6%) succeeded. These figures were consistent with the control group, where 6 (8%) of the 76 patients discontinued antidepressant use successfully. Patients who were recommended to discontinue their antidepressant medication reported a higher relapse rate than the control group (26% versus 13%, *P* = 0.05).

**Conclusion:**

Changing inappropriate long-term antidepressant use is difficult.

## How this fits in

Antidepressant use has increased, largely due to long-term prescriptions. Antidepressants are not very effective, especially in patients with depression of moderate severity. 

GPs are sometimes reluctant to withdraw inappropriate long-term antidepressant medication and long-term antidepressant users are frequently not motivated to stop antidepressant use.

## Introduction

During the 1990s, antidepressants were promoted widely and GPs were criticised for underdiagnosing and undertreating depressive and anxiety disorders.^1–3^ Efforts were made to increase quality of care, and prescription rates for antidepressants increased.^4^

Now, contrary concerns are raised concerning overtreatment with antidepressants.^5^ Long-term continuation contributes to the high level of antidepressant use.^6–10^ Studies suggest that many long-term users are exposed to antidepressants unnecessarily.^8,11,12^ One-third of long-term antidepressant users have been found to have no identifiable justification.^12^ In addition, a lack of medication review during the continuation of antidepressant treatment has been highlighted.^8^ Clinical guidelines recommend limiting the duration of antidepressants to 6 months after remission for a first or second depressive episode or a successfully treated anxiety disorder.^13–16^ The guidelines state that if after 4–6 weeks no remission has occurred, the medication should be switched to another antidepressant; if after another period of 4–6 weeks no remission has occurred, the guidelines advocate referral to a psychiatrist.^13–16^

Overtreatment with antidepressants is problematic. The effectiveness is questionable: about five of every six antidepressant users do not benefit.^17^ From the GP perspective, it is important to discuss how patients can use their own resources to cope with their problems; providing medication might be counterproductive, as medication use may disincentivise a patient to find non-pharmacological solutions, thereby diminishing patient empowerment in a context where regaining control is essential for recovery.^18^

This study concludes that overtreatment with antidepressants is very prevalent and that a considerable proportion of long-term use has no clinical justification. As such, this study aims to reduce inappropriate long-term antidepressant use in general practice. The authors will evaluate the effectiveness of a recommendation to cease antidepressant treatment which is tailored to both the patient and the psychiatric diagnosis.

## Method

### Study design

The authors conducted a cluster randomised controlled trial in primary care.^19^ The original protocol consisted of an overtreatment and an undertreatment trial. This article reports on the overtreatment trial. A summary can be found at the Nederlands Trial Register (NTR2032) (http://www.trialregister.nl/trialreg/admin/rctview.asp?TC=2032).

### Selection of study subjects

The study was conducted in 45 general practices in the Netherlands between February 2010 and March 2013. GPs identified long-term antidepressant users in their prescription database. GPs excluded patients based on the exclusion criteria below.

### Inclusion and exclusion criteria

#### Inclusion criteria

1. Long-term antidepressant use (≥9 months). All antidepressants were included, except monoamine oxidase inhibitors.
2. Written informed consent.

#### Exclusion criteria

1. Current treatment in a psychiatric inpatient or outpatient clinic.
2. Appropriate use of long-term antidepressants according to the Dutch guidelines for depressive and anxiety disorders (that is, a history of recurrent depression [≥3 episodes] and/or a recurrent psychiatric disorder with at least two relapses after antidepressant discontinuation).
3. History of psychosis, bipolar disorder, or obsessive compulsive disorder.
4. Current diagnosis of substance use disorder, excluding tobacco, because of the necessity of specialised treatment.
5. Non-psychiatric indication for long-term antidepressant usage, for example neuropathic pain.
6. Hearing impairment and/or insufficient understanding of the Dutch language.

Age was not an exclusion criterion.

### Informed consent procedure

Patients received an information brochure, via their GP, stating the purpose of the study; namely, to improve the treatment of patients using antidepressants long-term and to give a patient-tailored treatment recommendation. Patients could consent by filling out a return slip. Consenting patients were contacted in order to check inclusion and exclusion criteria.

### Diagnostic procedures and trial allocation

Eligible patients underwent a structured psychiatric interview by telephone using the CIDI (version 3.0), conducted by trained interviewers.^20–23^ Patients without a current psychiatric diagnosis or another indication for continued use (for example, neuropathic pain or chronic pain) were allocated to the trial.

### Randomisation

To prevent contamination between intervention and control group, a cluster randomisation was performed with the general practice as the unit of clustering. Random assignment was executed after patient recruitment was concluded per practice; a practice was either an intervention practice or a control practice.

### Follow-up procedures

Over the course of a year, all patients were routinely followed up. After 1 year they underwent the CIDI again. The self-report questionnaire was repeated every 3 months during this year.

### Intervention

A patient-specific letter was sent to the GP with the recommendation to discontinue the antidepressant. Information was provided on antidepressant tapering and the discontinuation syndrome. A gradual tapering programme was recommeded.^19^ The GP invited the patient to discuss the recommendation. No treatment restrictions were imposed in case of a relapse or the onset of a new psychiatric disorder after discontinuation. A return slip was included, to ascertain the patient's intention to comply with the recommendation. When either the GP or the patient did not intend to comply, the reasons for this were requested. In the control group, GPs were unaware which patients participated in this study and continued usual care.

### Primary outcome

The primary outcome was the proportion of participants who successfully discontinued their long-term antidepressant use after 1 year. Successful discontinuation is defined as no antidepressant use during the preceding 6 months and the absence of a depressive or anxiety disorder during the 1-year follow-up, as assessed by the CIDI. All information about medication use was collected in the follow-up CIDI, as well as in self-report questionnaires. Missing and contradicting prescriptions were checked by contacting the GP.

### Secondary outcome

The severity of general distress and depressive symptoms was assessed by the Brief Symptom Inventory (BSI-53),^24^ and the Centre for Epidemiological Studies Depression Scale (CESD),^25^ at baseline and after 3, 6, 9 and 12 months' follow-up. Somatic comorbidity was assessed with the TiC-P questionnaire.^26^

### Sample size estimation

The prospective sample size estimation aimed to provide at least 85% power for two-tailed testing with a type-1 error rate of 5%. To account for the cluster-randomisation, an intraclass correlation of 0.05 was used. Assumptions with respect to recruitment and outcomes were difficult to estimate. A 20% discontinuation rate for the control and 50% for the intervention group was expected. Spontaneous non-adherence to antidepressants is found to be 25%,^27^ and it was expected that this rate would decline as treatment time elapsed. The expected discontinuation rate in the intervention group was based on a primary care benzodiazepines discontinuation study.^28,29^ An average Dutch general practice (2400 patients) has approximately 50–60 patients using antidepressants long-term,^30^ with one-third possibly doing so inappropriately.^8 ^This study's recruitment rate was based on the results of three GPs piloting patient recruitment in their practices. It was found that an average practice would be able to recruit three patients who fulfilled all of the inclusion criteria and none of the exclusion criteria. Assuming a dropout rate of 25%, the required sample size was calculated as 34 practices and 136 patients.

### Statistical analyses

Analyses were conducted in IBM SPSS Statistics (version 20). Outcome analyses were performed on an intention-to-treat basis. Patients with an unknown primary outcome were conservatively classified as not having discontinued the antidepressant medication. The secondary outcome measures were analysed using a mixed models procedure for repeated measures, thus accounting for any missing values.

## Results

**Figure 1.**
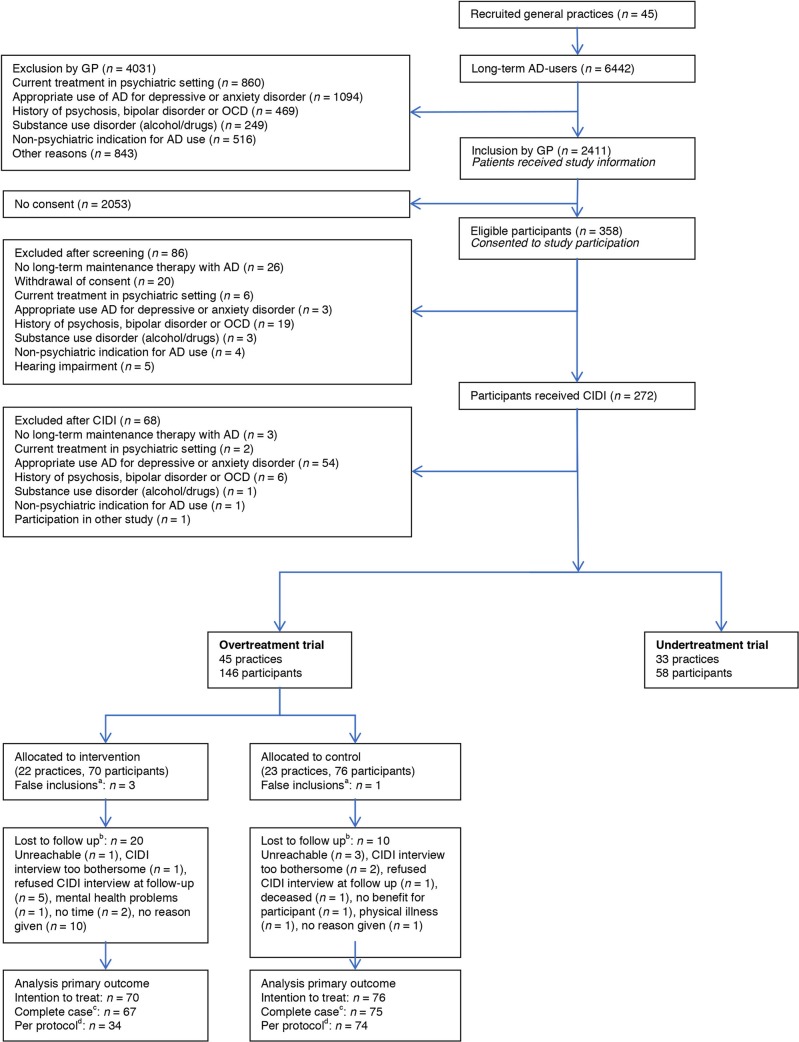
Flow diagram of practices and participants.^a^Post-randomisation patients did not meet inclusion criteria (human error during inclusion process). ^b^Patients who did not complete follow-up interview. ^c^Patients excluded with unknown primary outcome (due to dual primary outcome, excluded cases are less than patients lost to follow-up; that is, antidepressant use known via GP prescription database). ^d^Intervention group restricted to patients with the intention to comply to recommendation and patients excluded with unknown primary outcome.

Forty-five practices participated. In total, 6442 long-term antidepressant users were identified, of whom 2411 (37%) were deemed eligible by their GP. Of these patients, 358 (15%) consented to participate and 146 were included in this study (Figure 1).

### Study population

**Figure 2.**
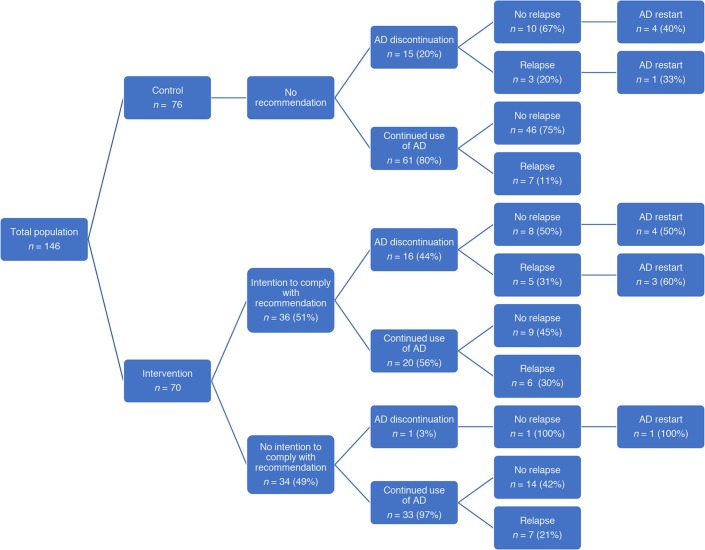
Patient flow and outcome in the overtreatment trial. In the intervention group 20/70 patients were lost to follow-up (12 in the group of patients with no intention to comply and 8 in the group with the intention to comply with the recommendation). In the control group 10/76 patients were lost to follow-up. AD = antidepressant.

Patient characteristics were well balanced at randomisation; any differences were not statistically significant (Table 1). Figure 2 shows the distribution of patients and their outcomes.

**Table 1.** Baseline characteristics of participants (inappropriate long-term antidepressant users) in the overtreatment trial at individual level in frequencies, unless stated otherwise. Overtreatment: ≥9 months antidepressant use, without a current indication for maintenance therapy

	Overtreatment trial, *n* (%)	
	Control (*n* = 76)	Intervention (*n* = 70)
Mean age, years (SD)	56 (14.3)	56 (12.9)
Male	24 (32)	20 (29)
**Marital status**		
Married or living together	60 (79)	56 (80)
Separated or divorced	0 (0)	2 (3)
Widow/widower	7 (9)	2 (3)
Single	9 (12)	9 (13)
**Lifetime psychiatric diagnosis**		
Any lifetime psychiatric diagnosis	48 (63)	53 (76)
Depression	35 (46)	39 (56)
Panic disorder or agoraphobia	13 (17)	13 (19)
Generalised anxiety disorder	13 (17)	22 (31)
Social phobia	20 (26)	16 (23)
**Antidepressant**		
Selective serotonin reuptake inhibitors	50 (66)	57 (81)
Serotonin–norepinephrine reuptake inhibitors	11 (14)	7 (10)
Other (non-tricyclic antidepressant drug)	10 (13)	2 (3)
Tricyclic antidepressant drugs	5 (7)	4 (6)
Median duration of antidepressant use at inclusion, years (range)	9.5 (1–56)	8.0 (1–48)
**Comorbidity**		
Cardiovascular disease	7 (9)	9 (13)
Cancer	6 (8)	8 (11)
Chronic obstructive pulmonary disease/asthma	12 (16)	9 (13)
Diabetes mellitus	11 (14)	3 (4)

SD = standard deviation.

In the intervention practices, the recommendation to discontinue was rejected in almost half of the cases (*n* = 34/70, 49%; 95% confidence interval [CI] = 37 to 60): by the patient in 14 cases (41%), the GP in 1 (3%), and as a shared decision in 16 (47%); in three cases data were missing. Reasons for rejecting the recommendation were as follows: fear of recurrence (*n* = 19, 56%); relapse after previous discontinuation (*n* = 4, 12%); presence of psychological symptoms (*n* = 5, 15%); wanting a second opinion (*n* = 4, 12%); and other, unspecified reasons (*n* = 2, 6%).

General distress or depressive symptoms at 3 months (approximately the time of consultation with GP to discuss the given recommendation) were not predictive for acceptance of the recommendation to discontinue (mean BSI 0.4, 95% CI = 0.2 to 0.5; mean CESD 17, 95% CI = 13 to 21 versus mean BSI 0.4, 95% CI = 0.2 to 0.6; mean CESD 15, 95% CI = 11 to 18).

### Primary outcome

In the intervention group, four patients (6%, 95% CI = 2 to 14) successfully discontinued their antidepressant use, in comparison to six patients (8%, 95% CI = 4 to 16) in the control group. When combining the intervention and control groups, successful discontinuation of antidepressant use was found in 10 patients (7%, 95% CI = 4 to 12).

### Secondary outcomes

The study found a marginally significant higher relapse rate in the intervention group (*n* = 18/70; 26%) compared to the control group (*n* = 10/76; 13%) (*P* = 0.05). Comparison of patients who continued their antidepressants identified a non-statistically significant higher relapse rate in the intervention versus control group (25% versus 11%, *P* = 0.07). This difference was not associated with antidepressant discontinuation.

Patients who successfully discontinued their antidepressant did not differ from the rest of the study population in sex, age, type of antidepressant used (selective serotonin reuptake inhibitors, tricyclic antidepressants or other) or psychiatric diagnosis. However, the mean duration of antidepressant use appeared to trend toward a shorter duration in patients who successfully discontinued their antidepressant (5.7 years; 95% CI = 1.6 to 9.7 years versus 9.6 years, 95% CI = 8.3 to 11.0 years, *P* = 0.077).

## Discussion

### Summary

This study shows the difficulty of discontinuing inappropriate long-term antidepressant use. Irrespective of the condition patients were allocated to, only 10 of the 146 patients with inappropriate long-term use of antidepressants (that is, use not recommended by clinical guidelines), were able to successfully stop in the year of the study. Half of the patients in this study rejected the recommendation to discontinue. Even when intending to comply, more than half (56%) ultimately did not. Interestingly, the number of patients in the control group spontaneously discontinuing their antidepressant was similar to the number of patients discontinuing their antidepressant in accordance with the recommendation.

A higher relapse rate was found in the intervention group. Strikingly, this was not associated with antidepressant discontinuation (Figure 2). Focusing on the use of the antidepressant could have caused a higher risk of relapse; patients could have felt obliged to act on this recommendation without an internal motivation, causing more anxiety about potential relapse and, consequently, a higher risk of actual relapse. In addition, it is possible that feelings of failure could arise when rejecting the recommendation, again resulting in a higher risk of relapse.

### Strengths and limitations

As far as the authors are aware, this is the first randomised controlled clinical trial focusing on long-term antidepressant use in patients in remission.

The studied intervention was based on previous experiences with discontinuation of long-term benzodiazepine use, where a stepped-care approach was found to be effective.^31^ A minimal intervention, consisting of an advisory letter or a consultation to discuss discontinuation with the GP proved effective to discontinue benzodiazepines.^32^ Apparently, the parallel with antidepressants was made too easily, with patients and GPs being hesitant to discontinue.

Of the large number of long-term antidepressant users, only a small portion consented to participate in this study (<15%). Patient recruitment is a known problem, especially in mental health research.^33^ However, despite the low response rate, the sample size to provide sufficient power for the trial was reached by approaching more practices and patients than originally anticipated. Due to privacy regulations, these long-term antidepressant users remain anonymous until giving consent. Unfortunately, it is not known why patients decided not to participate. It is conceivable that patients who were not willing to participate were more reluctant to change their antidepressant treatment. This would make the study's findings even more concerning as, in those circumstances, the chances are that with a larger, more generalisable population, the percentage of patients successfully discontinuing their antidepressant medication would even be lower. The recruitment success of patients for participation in such an evidence-based intervention could illustrate the difference between perceived self-interest (by the patient) and perceived patient interest (by researchers and practitioners). Further studies about antidepressant discontinuation should therefore focus on patients who are motivated for discontinuation.

Due to the pragmatic nature of this study, the intervention was not imposed on the patients and their GPs. Noncompliance with the given recommendation was found in almost half of the cases. Further qualitative research might be helpful to understand the barriers patients and GPs perceive in discontinuing long-term antidepressant use in patients in remission, and to facilitate the construction of a more effective intervention to reduce inappropriate long-term antidepressant use.

### Comparison with the literature

This study showed that many patients reject a proposal to discontinue antidepressant use, that many GPs agreed with the decision not to follow the advice to discontinue and that a large number of patients (32/36) who agreed to follow the protocol failed to do so. Clearly, there is a large gap between what guidelines recommend and what happens in daily practice. Although deviation from a guideline may be consistent with good care, the scale of non-adherence raises another possibility; namely, that apprehensiveness about change and difficulties with discontinuation is much more important than was initially expected. This apprehensiveness about change was found in both patients and GPs. Qualitative research has suggested that patients attribute their wellbeing to the (continued) use of antidepressants. They are more afraid of stopping than of continuing, taking a 'better safe than sorry' approach.^34–35^ They believe their condition to be chronic and requiring life-long treatment, while feeling uncomfortable with this prospect.^34–36^ GPs also perceive barriers to discontinuation, wishing 'not to disturb the "equilibrium" the patient experiences', to 'follow the path of least resistance' and to 'let patients be'.^36^ GPs operate in a difficult environment: dealing with guidelines, their own fears, patients’ opinions and fears, and the difficult process of discontinuation. Adherence to guidelines is difficult. Attempts to discontinue antidepressant use — while very desirable in the light of the huge prescription rates — become a complex task when taking all these factors into account.^37^

Lately, guidelines have become more conservative in their recommendations concerning the prescription of antidepressant medication. It is conceivable that the GPs in the PANDA study did not agree with the recent guidelines. Alternatively, it may be that GPs had to get used to these new insights, with guidelines having previously advocated for increased antidepressant prescription. The prescribing behaviour of GPs is certainly an important topic: they should become more reluctant in prescribing, and should inform patients that the medication will only be necessary for a limited period of time and can be discontinued after being in remission for a period of 6 months. Patients discontinuing their antidepressant medication should also receive more information, guidance and support than they receive at present. In addition to the issue of the difficulty of discontinuing inappropriate antidepressant medication, there are several other reasons justifying a reluctance to prescribe antidepressant medication. Firstly, the evidence of the substantial placebo effect in patients with depression is strong, with the exception of the more severe cases.^38^ Secondly, the availability of psychological treatments suitable for primary care is growing. Important examples are problem-solving treatment and behavioural activation; both treatments can be delivered by junior mental health workers.^39^

### Implications for practice

This study demonstrates the difficulty of correcting unnecessary (according to the guidelines) long-term antidepressant use, fuelled by an apprehensiveness regarding change on the part of both patient and GP. A recommendation to discontinue is not effective, and maybe even counterproductive. The authors advocate developing education programmes for GPs, including such topics as GPs’ attitudes towards discontinuation, appropriately motivating patients to discontinue antidepressant use, and managing the process of discontinuation. Notwithstanding, it is felt that the first, and possibly most important, step to prevent inappropriate long-term use of antidepressant medication in primary care is to be more restrictive in prescribing antidepressant medication in the first place and make more use of alternative, non-pharmalogical treatments. It might be useful to forewarn patients about the difficulty of discontinuing and to encourage using antidepressants only for a limited period. Regular review could possibly prevent both overtreatment and undertreatment.
